# Savings in per-passenger CO_2_ emissions using rail rather than air travel in the northeastern U.S

**DOI:** 10.1080/10962247.2020.1837996

**Published:** 2021-09-23

**Authors:** C. Andrew Miller

**Affiliations:** U.S. Environmental Protection Agency, Office of Research and Development, Research Triangle Park, NC, USA

## Abstract

Individuals and institutions seeking to reduce travel-related carbon dioxide (CO_2_) emissions by changing travel modes need information on the amount of CO_2_ that can be saved by rail travel rather than air travel. This study uses flight emissions data from the International Civil Aviation Organization (ICAO) to estimate average per-passenger CO_2_ emissions saved by using rail travel between selected city pairs in the northeastern U.S. Trend lines are developed from the ICAO data for different aircraft types to facilitate comparison with CO_2_ emissions from rail travel. Separate rail emission factors are calculated for portions of Amtrak’s system operating electric and diesel locomotives. An adjustment factor is estimated to account for longer rail distances than flight distances. Results show rail travel has generally lower CO_2_ emissions than air travel, with substantially lower emissions for electrified segments of the Amtrak system. At flight distances of over 700 miles, air travel using single-aisle jets can have lower per-passenger CO_2_ emissions compared to diesel-powered rail travel, accounting for the longer distances by rail.

**Implications:**

Savings in per-passenger CO_2_ emissions using rail rather than air travel in the northeastern U.S. Travel by rail in the northeastern U.S. results in lower CO_2_ emissions compared to travel by air between the same city pairs using existing airline and passenger rail infrastructure. Savings are higher for cities connected by electrified rail.

## Introduction

Individuals and institutions are becoming increasingly interested in understanding the climate impacts of their activities. Air travel in particular has received considerable attention for its greenhouse gas (GHG) emissions, particularly carbon dioxide (CO_2_). This is in part due to past growth in air travel and projections for continued growth, combined with a lack of low-carbon flight options. The International Civil Aviation Organization (ICAO) projected an annual average growth rate of 4.3% in commercial air transport through 2035 as measured by available tonne-kilometers (Fleming and de Lépinay 2019). The U.S. Federal Aviation Administration’s base-line projection for 2040 anticipated a 50% increase in miles flown by domestic U.S. air passenger carriers (Federal Aviation Administration 2020), and the U.S. Energy Information Administration projected a ten-fold increase in U.S. air transport seat-miles between 2019 and 2050 (Energy Information Administration 2020). These projections reflected long-term trends in global air travel and did not anticipate the sort of large-scale disruption now occurring due to the COVID-19 pandemic.

Low-carbon flight options remain scarce, at best. Even under the ICAO’s most optimistic scenario, research suggests that the airline industry is unlikely to meet the organization’s aspirational goal of a 2% per year improvement in fuel efficiency (and therefore CO_2_ reductions) (Fleming and de Lépinay 2019). The Air Transport Action Group (ATAG) claims the industry is achieving the 2% annual efficiency improvement, as they work toward a 2050 goal of reducing total emissions by 50% compared to 2005 (ATAG 2020). In the U.S., domestic air carrier fuel efficiency as measured in seat-miles per gallon has increased by 57% since 1990 (Bureau of Transportation Statistics 2020); however, this is only an average 1.6% increase per year. Thus, the ICAO aspirational goal is not substantially more ambitious than past efficiency improvements and would result in increasing CO_2_ emissions from aviation in any case. In short, the outlook for low-carbon air travel appears at best limited in the near term.

As we are learning through the massive shift to remote interactions in response to the COVID-19 pandemic, physical presence may not be as necessary as we had previously assumed. The most effective means of reducing travel-related emissions is to avoid traveling, but this option is not always appropriate or even viable. Thus, those individuals and institutions who need to travel and seek to reduce their travel-related CO_2_ impacts in the near term are left with two options: (1) purchase offsets [i.e., GHG reductions at other locations of an amount equivalent to the given flight’s emissions, so that the flight’s emissions are “offset” by the remote reduction (U.S. Environmental Protection Agency 2018)]; or (2) use a different mode of travel that emits less GHGs. In both cases, the traveler or institution needs information on the average GHG emissions per passenger for a given flight, and, when changing mode of travel, the emissions associated with the alternative travel mode.

There is no shortage of flight emission calculators – they are offered for free public use by airlines, news media, advocacy groups, and a few by governmental organizations. Information on trip-level emissions data for alternative travel modes is less common. EcoPassenger offers a CO_2_ savings calculator for European rail trips (EcoPassenger 2020) and CN (formerly the Canadian National Railway) provides a carbon calculator for freight (CN 2020). Amtrak provides an opportunity to purchase offsets for 5 USD (3000 miles), 10 USD (6000 miles) or 20 USD (12000 miles), but does not provide estimates for emissions or offsets between city pairs (Amtrak 2020c).

The purpose of this study is to provide more detailed information about the amount of CO_2_ saved when traveling by rail rather than air between cities in the northeastern U.S. The study uses readily available data sources to examine the difference in CO_2_ emissions when using rail rather than air travel between city pairs on current Amtrak routes within about 400 flight miles from Washington, DC, and with more than 500,000 annual airport arrivals and departures. The analysis focuses on the northeastern U.S., which has the most intensive intercity passenger rail operations in the U.S., with 12 of the 15 busiest stations nationally. The analysis is limited to intercity trains operated by Amtrak, the brand name of the federally owned National Railroad Passenger Corporation that operates an average of 300 intercity trains each day to more than 500 stations (Amtrak 2019).

Unlike many studies that examine potential changes that could be achieved through different policy alternatives, the results presented in this study reflect existing equipment and travel routes. In addition to providing individuals and institutions with information on the routes for which rail travel can currently be expected to provide CO_2_ savings compared to air travel, these results also identify important factors that determine the extent of those savings, including availability of electrified rail and differences in travel distance between air and rail transport. Needs for improved data, especially for route-specific emissions from passenger rail operation, are also presented.

## Research background

A considerable body of work has been published regarding potential CO_2_ reductions that can be achieved by replacing at least a portion of air travel with high-speed rail (HSR). These studies have largely focused on evaluating the potential GHG reductions related to shifting intercity travel to HSR from air travel. Dalla Chiara et al. (2017) simulated HSR and aircraft operations to compare energy requirements for both modes. They focused on travel in Italy and estimated energy consumption per seat-mile for HSR to be less than that for air travel, by at least an order of magnitude. Prussi and Lonza (2018) compared CO_2_ emissions of air travel and HSR between seven European city pairs. They found net CO_2_ savings by switching from air to rail travel for all seven routes in two future scenarios with increased reliance on rail.

Other studies compared air travel to HSR, largely in the context of competition between the two, and with a focus on potential future adoption of HSR (Albalate and Bel 2012; Behrens and Pels 2012; Chester and Ryerson 2014; D’Alfonso, Jiang, and Bracaglia 2016; Janic 2003; Zhao and Yu 2018) Other work has evaluated intercity transportation system design and performance more generally. Krishnan et al. (2015) described a long-term transportation investment model that included both transportation and electric infrastructure in the U.S., with a focus on high-speed rail. Socorro and Viecens (2013) presented an evaluation of an integrated air-rail transportation network, focusing on Spain. Yang et al. (2009) discussed the role of changes in the California transportation system in meeting an 80% GHG reduction target.

In general, these studies found that high-speed rail provided environmental benefits, including GHG reductions, over other travel modes, with two important caveats. First, rail cannot provide these benefits if it cannot provide service competitive on price and travel time compared to other travel modes. Several studies noted that rail travel was more likely to be used than other modes at distances of 100–500 miles (160–800 km), with some studies indicating that rail travel could be competitive with air travel at distances of up to 600 mi (1000 km) (Albalate and Bel 2012; Chen 2017; Dalla Chiara et al. 2017; Prussi and Lonza 2018).

Second, rail and air travel have significantly different infrastructure requirements that have important implications for environmental impacts over the life cycle of each mode. Chester and Horvath (2010) looked at life cycle impacts of HSR in California, and Robertson (2016) evaluated the difference in life cycle emissions for air and HSR travel in Australia. Both studies found that rail can have long-term benefits over other travel modes, but that high rail occupancy levels were crucial to achieving those benefits.

The studies cited here provide a glimpse at the substantial body of literature on high speed rail, particularly focused on questions about the development and potential effectiveness of HSR policies. However, they are considerably less relevant to understanding the difference in CO_2_ emissions attributable to a single passenger choosing between air and rail travel with existing systems. Baumeister (2019) and Baumeister and Leung (2020) developed an analysis that is the most immediately relevant to this question. They examined CO_2_ equivalent (CO_2_eq) emissions per passenger between 16 city pairs in Finland and showed substantial savings when traveling by rail compared to air, bus, or passenger car between the same cities. Borken-Kleefeld, Berntsen, and Fuglestvedt (2010) compared CO_2_ emissions from different travel modes based on existing European equipment and infrastructure, with a focus on identifying key parameters that affect emissions from different modes. But no published study has been found that compares emissions from air travel to those from rail travel for the northeastern U.S., the region with the greatest availability for passenger rail transport in the U.S., based on number of stations and frequency of service.

## Methodology

This study seeks to directly compare CO_2_ emissions in mass per person per trip for air travel and rail travel between a given pair of cities. Data in this form are available from multiple sources for air travel, with varying levels of detail. Comparable data are not available for rail travel, which need to be developed using data from several sources. For both air and rail travel, the distances traveled are also important to gaining a more complete understanding of the emission differences between the two modes. This section will discuss the approaches taken to select the city pairs evaluated, the sources of data, and the calculations needed to obtain comparable CO_2_ emissions data for travel between city pairs.

It is important to recognize that CO_2_ emissions are not the only consequence of air travel that affects radiative forcing (Jungbluth and Meili 2019). Aircraft-induced clouds can have a larger near-term radiative effect than CO_2_ (Kärcher 2018) but cannot be characterized in general terms that will allow direct comparison to other sources, specifically rail in this instance. This means that comparisons based only on CO_2_ will underestimate the radiative forcing associated with air travel.

### City pair selection

Within the region loosely defined here as the north-eastern U.S., initial selection of city pairs was determined by four factors: (1) within 500 air miles of Washington, DC; (2) location on an Amtrak route; (3) more than 500,000 annual passengers at each city’s airport; and (4) at least one regularly scheduled airline flight between the two cities. For this analysis, cities were selected that met the four criteria above and were also included in the Federal Railroad Administration’s High Speed Intercity Passenger Rail Program in the Northeast and Southeast Regional Investment Plans (Federal Railroad Adminstration 2019). These factors resulted in selection of 16 cities, from Portland, ME in the north to Charlotte, NC in the south, and from Boston in the East to Buffalo and Pittsburgh in the west. Cincinnati, OH was also added to the analysis as an example of a city that is characterized by much longer rail distances than air distances.

**Figure 1.**
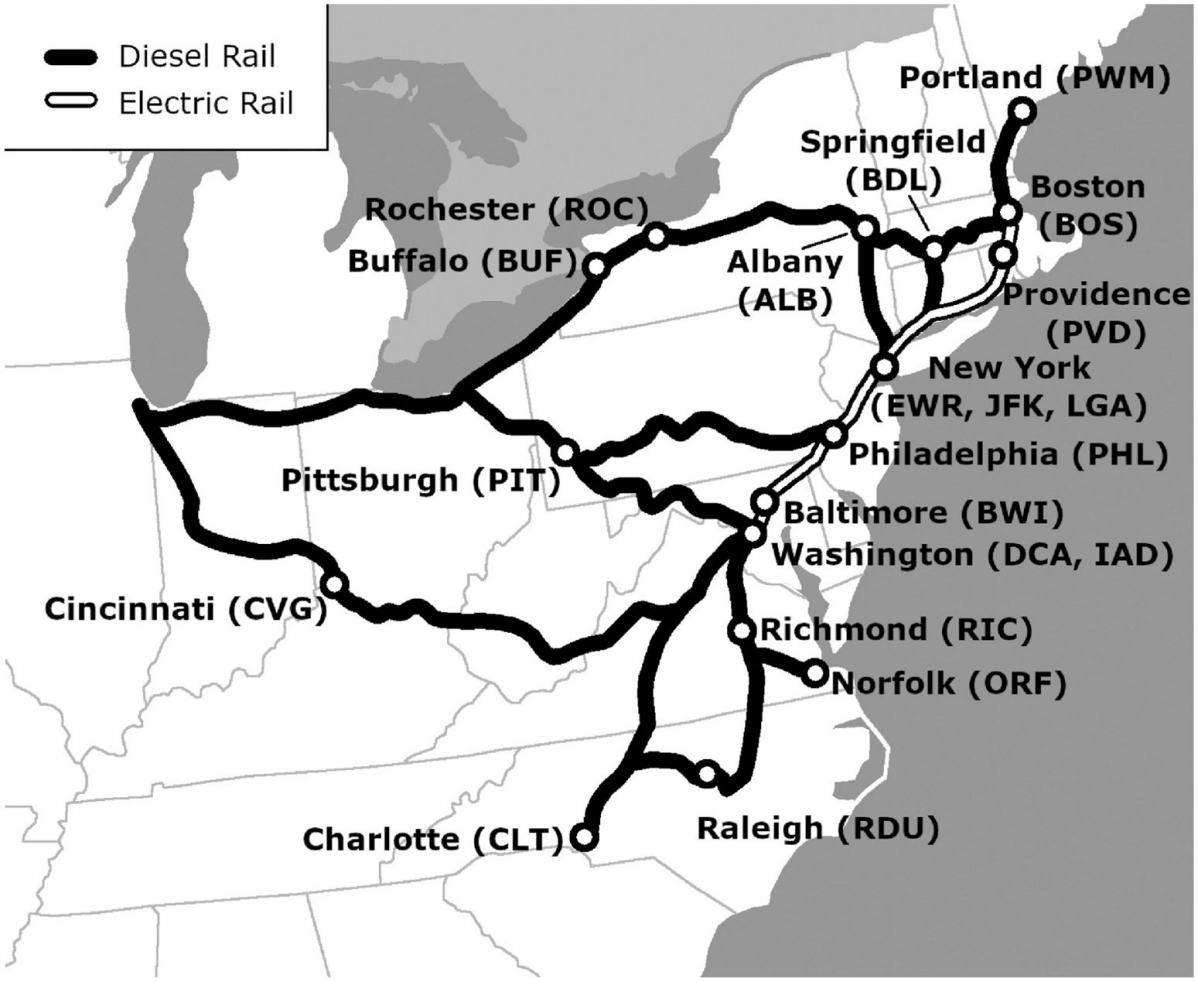
Cities, airports (airport codes in parentheses) and Amtrak routes analyzed. Adapted from Amtrak.

**Figure 2.**
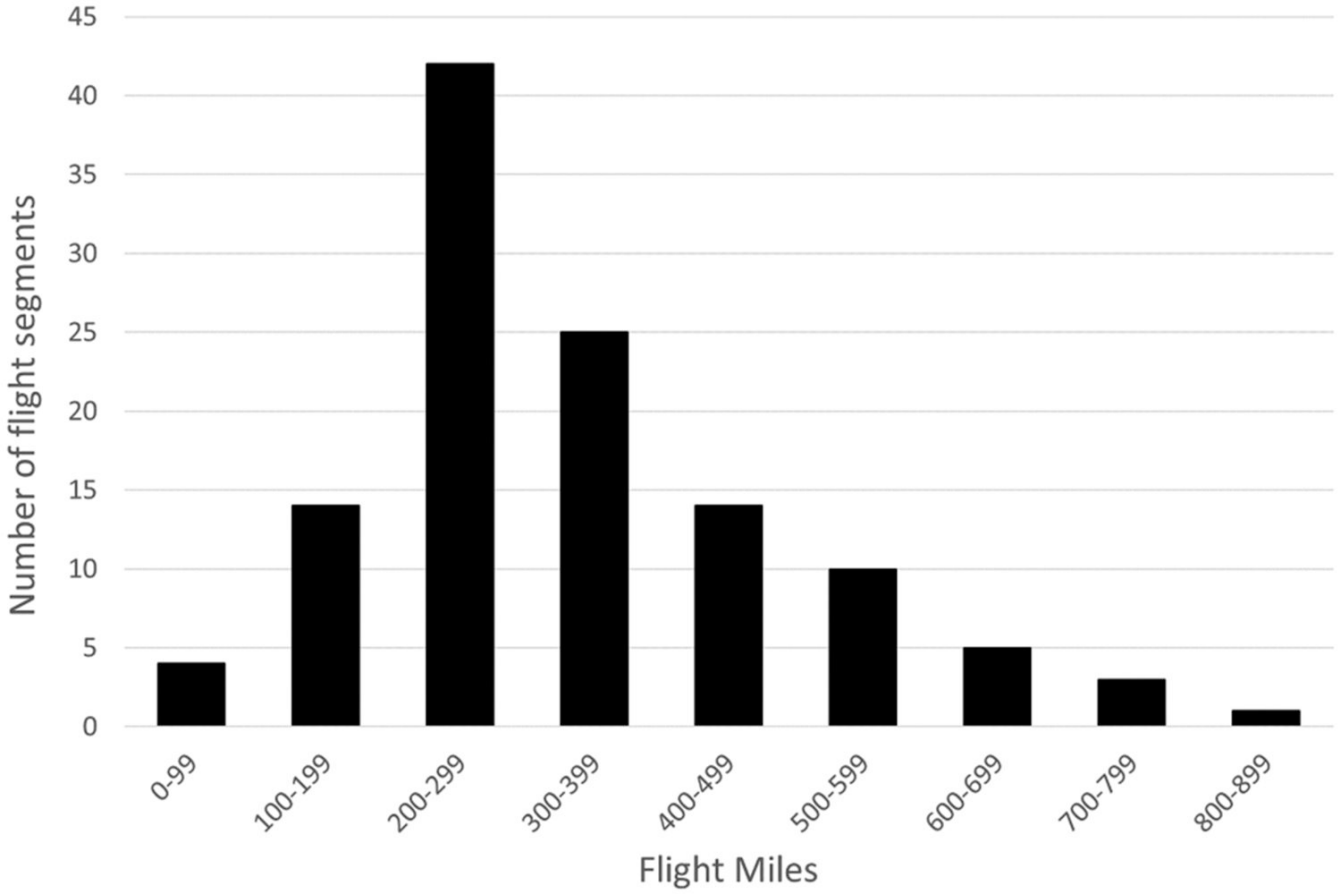
Number of flight segments by length.

The 17 cities have 20 airports, with three serving the New York metropolitan area, and two serving the Washington metropolitan area. Table 1 and Figure 1 show the 17 cities and their associated airports. There are 118 airport pairs with direct flights between them, ranging in distance from 90 to 811 miles. Just over half (60) of the flight distances were less than 300 miles, as shown in Figure 2. The selected flight segments reflect data collected in early 2020 and do not reflect changes in aircraft type or flight schedule that may have occurred as airlines responded to air travel reductions due to COVID-19 concerns. The data also do not reflect the seasonal nature of some routes. While these factors may change the results for specific routes, they are not expected to change the overall differences between rail and air travel emissions.

### Travel distance data

There is generally only a single rail route between city pairs, but there may be multiple air routes given the availability of connecting flights. Because the rate of fuel burn is much higher during takeoff and climb to cruise altitude, air travel CO_2_ emissions are strongly influenced by the number of landing and takeoff (LTO) cycles (Miyoshi and Mason 2009). For emissions from air travel to be as directly comparable to those from rail travel as possible, only direct flights between city pairs were included in the analysis. City pairs without direct flights were not included.

While rail emissions are higher during acceleration following a station stop, the effect on total trip emissions is much smaller than the analogous landing and takeoff emissions for air travel. Thus, the shortest rail distance between each city pair was used, regardless of whether one or more changes of train would be required. In practice, changing trains would be an inconvenience for individual travelers and would likely result in less use of rail.

The factors related to mode choice are beyond the scope of this study but are important to recognize. These factors include door-to-door travel time for each mode, which will depend upon the specific circumstances of travel end points, weather, and even the time of day or season when the trip is taken as they strongly affect congestion to and from airports and train stations. Total travel cost, passenger comfort, security restrictions, and other factors also influence mode choice, which will ultimately affect system-level emissions (Albalate and Bel 2012).

This comparison accounts only for point-to-point travel between terminals in each city pair and does not include emissions associated with travel to and from the train station or airport, airport or rail operations and maintenance, or life cycle emissions associated with infrastructure construction and operation, vehicle manufacturing, or fuel extraction, processing, and transport.

Rail distances are taken from current Amtrak time-tables (Amtrak 2020b). Flight distances are great circle distances (i.e., the shortest distance between two points on a sphere) between selected airports and are taken from the ICAO Carbon Emissions Calculator (ICAO 2016). Detours due to weather or flight traffic or deviations from great circle routes caused by following FAA Jet Routes between airports are not considered, although the ICAO calculator does account for an average deviation between great circle distances and actual flight distances (ICAO 2018).

### Flight CO_2_ emissions data

Emissions of CO_2_ from aircraft are generally derived from consumption estimates for jet fuel. Fuel consumption depends upon the flight distance and profile, load, and aircraft type. The flight profile includes rate of climb, cruise altitude, and rate of descent, and is relatively stable for a given flight path between given airports. Aircraft load includes number of passengers and associated luggage as well as separate air cargo that may be included on a given flight. Aircraft type varies by airline, and for flights of 500 miles or less include either single-aisle jets (e.g., Boeing 737 and 717 series aircraft and Airbus 320 series aircraft, also referred to as short-haul or narrow-body aircraft) or regional jets (e.g., Bombardier CRJ series aircraft and Embraer air-craft). In general, regional jets have fewer than 100 seats and are used for shorter flights and for city pairs with lower traffic levels. On a per-passengermile basis, single-aisle jets are generally more fuel (and therefore, CO_2_) efficient than regional jets (Graver, Zhang, and Rutherford 2019), although choice of aircraft to a given route depends upon the number of passengers traveling between city pairs and airport configuration, among factors other than emissions. Baumeister (2017) also noted discrepancies between different calculators in both methodology and results and chose to use flight-specific data for load factors, passenger-to-freight factors, and supplied seats to develop fuel consumption and CO_2_ emission estimates for those flights. While this approach is presumably more accurate for a given flight, it is questionable whether it is more accurate for the average emissions over all flights on a given route.

There are numerous carbon emission calculators available for public use, but there is strikingly little consistency among them. This problem was recognized in 2009 and seems to have changed little since that time. Miyoshi and Mason (2009) noted that the same journey “would be measured at different levels of carbon emissions by different calculators,” and that the different calculators appeared to adopt different estimation methodologies.

Flight emissions are taken from the ICAO Carbon Emissions Calculator, using one-way flights and economy-class estimates. The methodology used by the ICAO calculator is well documented and represents the average emissions by the specific mix of aircraft types operating between two airports as well as the average load factor (actual number of passengers divided by the number of available aircraft seats). The ICAO estimate is based upon the fuel burned by the average aircraft between two airports, with reductions from the total to account for non-passenger air freight to more accurately reflect emissions directly associated with passenger travel. The estimate also accounts for different service classes (e.g., first, business, standard) and the different aircraft space requirements and thus, emissions, associated with a seat in each class. Because it is based on the average aircraft fuel consumption between city pairs, the ICAO estimate implicitly includes taxiing and other ground movements of aircraft as well as consumption due to rerouting due to weather or air traffic. The calculator methodology thus accounts for greater susceptibility to increased emissions at specific airports or flight segments in its city pair estimates.

The U.S. Environmental Protection Agency (EPA) includes rail and aircraft emissions in their Simplified GHG Emissions Calculator developed for use by small businesses to estimate their annual GHG emissions. The EPA calculator is a downloadable spreadsheet and uses average emission factors for aircraft based on three flight length of the other carbon emissions calculators examined, only one provided as much information as the ICAO calculator. The Atmosfair emissions calculator allows users to specify the specific aircraft type, shows average emissions for the flight segment, and is backed by good documentation (Brockhagen, Höhne, and Schultz 2011). The Atmosfair emissions tended to be higher than those reported by the ICAO calculator. However, in the absence of a thorough, independent comparison of the two calculators, the ICAO calculator was selected for this analysis, for three reasons. First, as the international body responsible for international civil aviation rules and coordination, it represents the most authoritative source of information. Second, it is updated on an annual basis to reflect changes in types and numbers of aircraft used between city pairs, although passenger load factors and passenger-to-freight factors are not (these data were last updated in 2016) (ICAO 2018). And third, it provides a list of aircraft types that are accounted for in the emissions estimates.

The ICAO calculator presents CO_2_ mass for a single passenger, flight distance, and aircraft types used between each airport pair. CO_2_ mass was divided by flight distance to determine lb CO_2_ per passenger-mile for each airport pair.

### Rail CO_2_ emissions data

CO_2_ emissions from rail transport come from two sources: direct combustion of diesel fuel in diesel-electric locomotives, and emissions from the generation of electricity used in electric locomotives. Unlike flight emissions, there are no readily available emissions calculators for rail travel in the U.S. Amtrak provides some information on relative energy use in a graphical display of energy use per passenger mile compared to other forms of travel (Amtrak 2020c). However, this does not distinguish between diesel and electric motive power or account for factors such as train length, number of passengers, speed of travel, intermediate station stops, or track grade.

The lack of more detailed data means that system-level information will need to be used. Amtrak reports diesel fuel used in revenue operation across their system (Amtrak 2018). The Bureau of Transportation Statistics (BTS) Rail Profile (Bureau of Transportation Statistics 2019) provides annual data for electricity consumption by Amtrak locomotives and passenger-miles traveled on Amtrak. Given that CO_2_ per gallon of diesel fuel is a straightforward calculation, this leaves two factors to be determined – the CO_2_ emissions from electricity generation and the allocation of passenger-miles to diesel- and electric-powered routes.

Amtrak uses electric locomotives primarily between Boston and Washington, DC, so the diesel emission factor can be used for the remainder of the system with the exception noted below. For this analysis, it is assumed that any travel between stations that lie between Boston and Washington will be powered by electric locomotives, and travel between any stations that do not lie along that route will be powered by diesel locomotives. The BTS data show Amtrak locomotives used 485 million kWh of electricity in 2018, all of which is used between Boston and Washington and between Philadelphia and Harrisburg, PA (Amtrak 2020a).

In this analysis, it is assumed that the electricity used for electrified portion of the Amtrak system is generated within the states through which the electrified rails run (Connecticut, Delaware, Maryland, Massachusetts, New Jersey, New York, Pennsylvania, Rhode Island, and the District of Columbia). The EPA reports electricity generation and associated emissions by state, which allows calculation of an average emission factor for electricity along the electrified portion of the system (U.S. Environmental Protection Agency 2020). The EPA data show these states generated a total of 548 million MWh with 175 million short tons of CO_2_ in 2018, resulting in an average emission factor of 0.640 lb CO_2_/kWh across the nine states. With the BTS estimate of 485 million kWh used by Amtrak locomotives, this yields an estimated 155,000 short tons of CO_2_ from Amtrak electric locomotive operations in 2018.

Diesel emissions are calculated by multiplying the revenue locomotive diesel fuel consumption reported by Amtrak (50.3 million gallons) by an average of 22.40 lb CO_2_ per gallon (Energy Information Administration 2016) to obtain 563,000 short tons of CO_2_ from Amtrak diesel revenue operations in 2018. Recall that while the CO_2_ related to electric locomotive operation occurs only between Boston and Washington, Amtrak’s CO_2_ emissions from diesel locomotives occur across the country.

To be able to compare emissions between city pairs, emissions per passenger-mile need to be determined by allocating to the portions of the routes that are electrified or not. This is made somewhat more straightforward by the fact that passenger-mile values can be determined by statistics published by the Rail Passenger Association (RPA) for each of Amtrak’s routes (Rail Passengers Association 2020). RPA publishes annual fact sheets for each of Amtrak’s 47 routes, including its Northeast Regional Service and its Acela Express route. Both operate between Boston and Washington and are powered by electric locomotives. The RPA publishes total passengers and average trip length for each route, which provides total route-level passenger-miles.

Eleven routes partially operate over the electrified portion of the system, usually traveling from New York or Boston and continuing south from Washington. These trains switch from electric to diesel locomotives at Washington before continuing south. To estimate the passenger-miles for the electric and diesel portions of the route, the fraction of passenger-miles powered by electric locomotives is assumed to be the same as the fraction of track distance that is electrified.

The RPA fact sheets report a total of 6.34 billion passenger-miles traveled on Amtrak in 2018 (compared with 6.36 billion passenger-miles reported by BTS, a difference of 0.3%). The Northeast Regional Service and Acela Express accounted for 1.96 billion passenger-miles, and the fraction of the other 11 routes that operated over the electrified portion of the Amtrak system added an additional 0.22 billion passenger-miles, for a total of 2.31 billion passenger-miles on the electrified portion of the system. Using the RPA data for consistency, this results in 4.03 billion passenger-miles for the diesel portion of the system.

Using the CO_2_ emissions from each energy source calculated above yields an emission factor of 0.280 lb CO_2_/passenger-mile for diesel locomotives and 0.134 lb CO_2_/passenger-mile for electric locomotives. These results showing electric locomotives emit less than half the emissions of diesel locomotives per passenger mile emphasize the importance of rail power type to CO_2_ emissions. Because there is no source of data on route-specific emissions, the average emission factor for the Amtrak system in the northeastern U.S. is calculated by assuming the ratio of passenger-miles on electric-powered Amtrak routes to passenger-miles on diesel-powered Amtrak routes is the same as the ratio between miles of electric-powered routes to the miles of diesel-powered routes. This allows an estimate of total CO_2_ emitted from each of the electric and diesel portions of the route. These are then added, and the total divided by the total passenger-miles traveled to obtain an average emission factor of 0.227 lb CO_2_/passenger-mile for this analysis.

## Results

### Emissions by aircraft type

**Figure 3.**
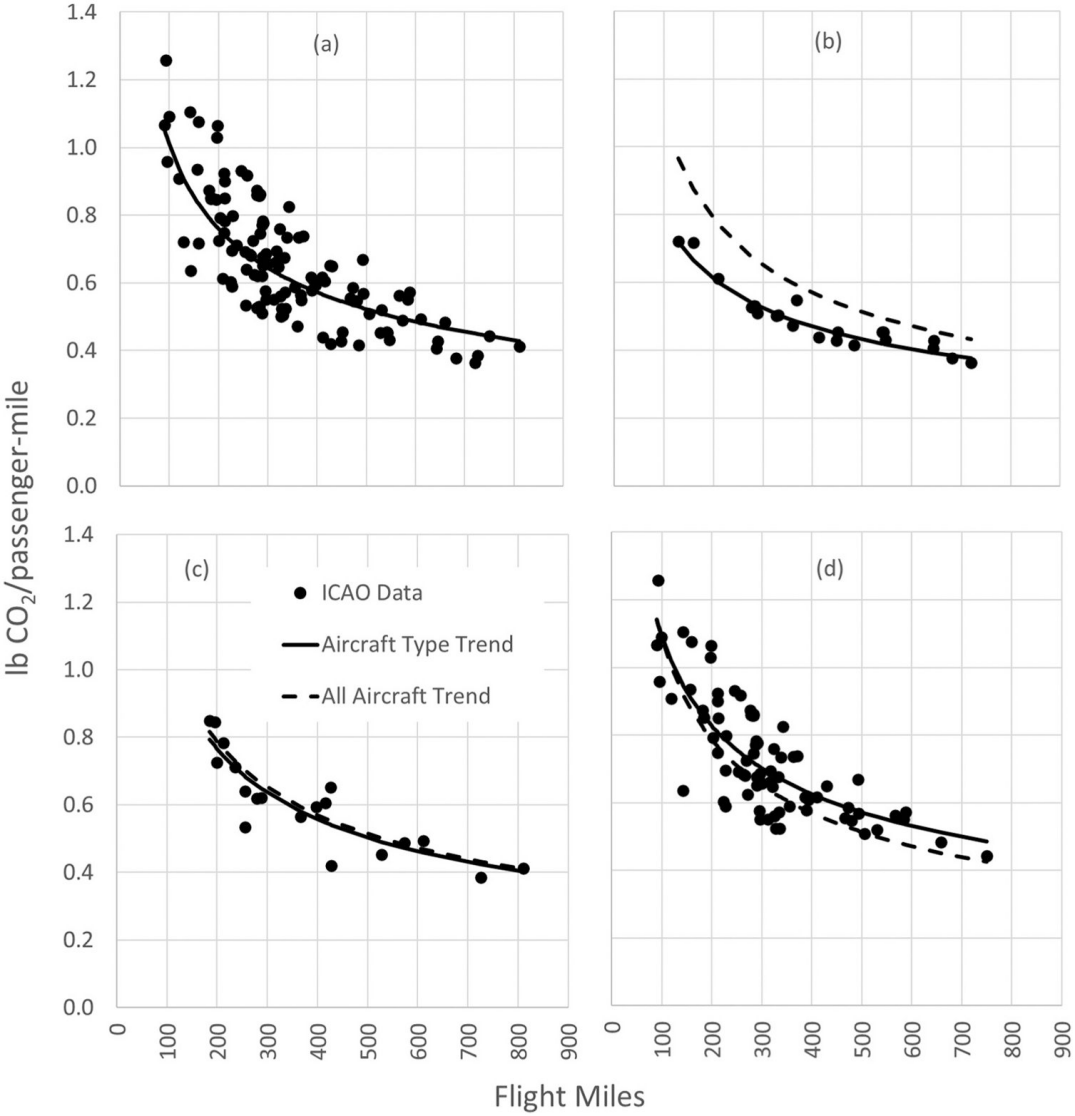
ICAO emission factors and emission factor trends, in lb CO_2_ per passenger-mile, by flight distance for (a) all aircraft; (b) single-aisle jets; (c) mixed single-aisle and regional jets; and (d) regional jets.

To facilitate the comparison of emissions between air and rail travel, it is useful to estimate the average emission factor as a function of flight distance. In this analysis, aircraft were divided into two categories – single-aisle jets and regional jets. None of the analyzed city pair flight segments used twin-aisle (wide body) aircraft. Only one flight route (Boston to Portland, ME) indicated use of turboprop aircraft, which did not allow for meaningful analysis by aircraft type; thus, no turboprop aircraft are included in the analysis. Nineteen airport pairs were served by single-aisle jets only, 34 by a mix of single-aisle and regional jets, and 65 by regional jets only. Figure 3 shows the emissions-distance relationships for all aircraft, single-aisle jets, mixed single-aisle/regional jets, and regional jets. The ICAO data change linearly with distance on a log-log scale, whether aggregating data from all routes analyzed in this study or when distinguishing routes by the three aircraft categories.

The route-specific ICAO data are shown along with the estimated curves. The curve for total aircraft is plotted with the curves for the aircraft types as comparison. The only type that shows a distinct difference from the total emission–distance curve is for single-aisle jets. This is reasonable, given the considerably fewer routes served exclusively by single-aisle jets. It is also reasonable that the curve is lower than that for total aircraft, as the larger single-aisle jets are generally more fuel efficient on a per passenger-mile basis than regional jets.

### Flight miles vs. rail miles

**Figure 4.**
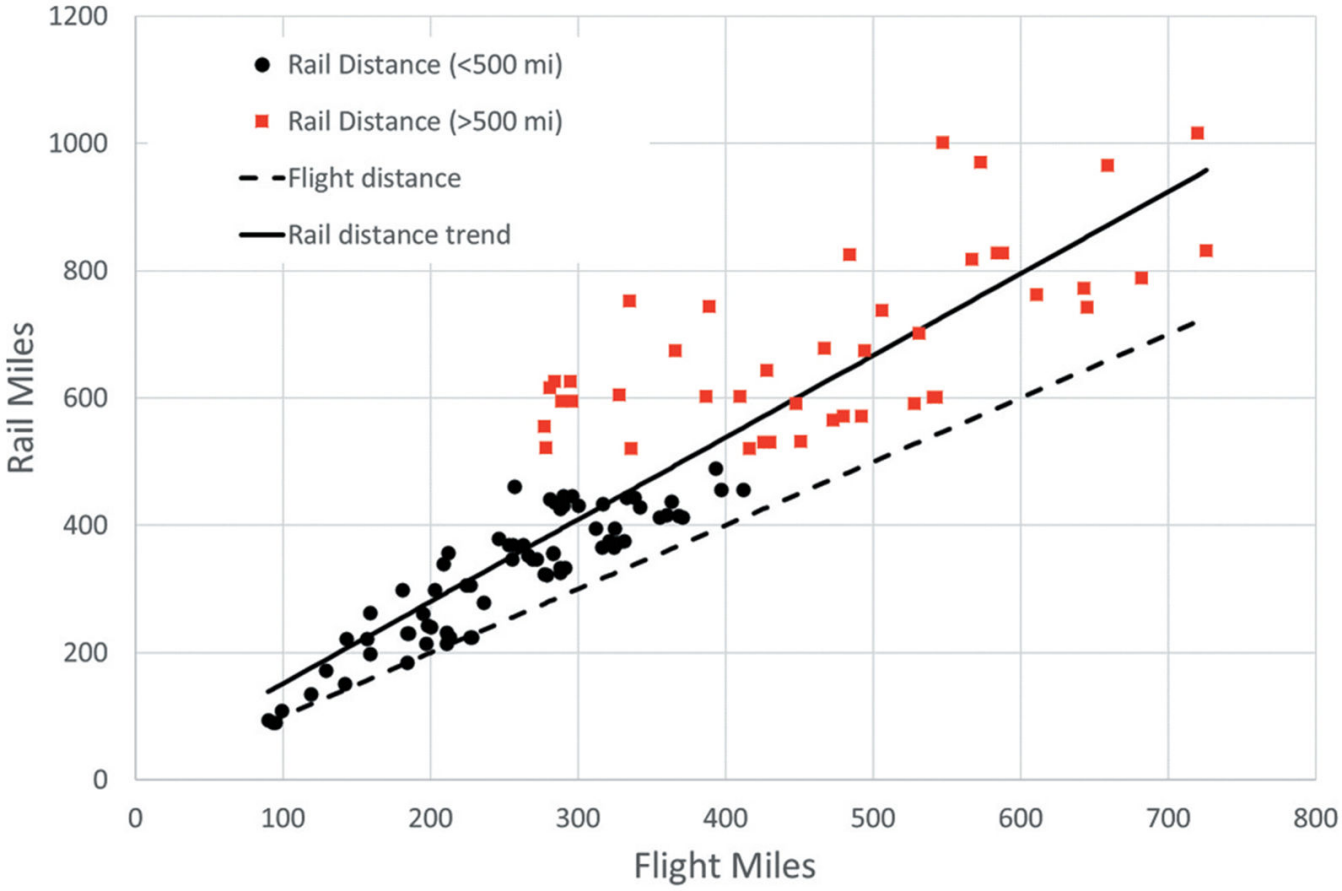
Comparison of rail miles to flight miles for the 120 city pairs analyzed. Trips with rail distances greater than 500 miles are indicated by red squares.

Before comparisons of per-passenger CO_2_ emissions from air and rail travel can be made, the difference between air miles traveled and rail miles traveled must be considered. As Prussi and Lonza (2018) noted, rail miles are generally longer than flight miles. This is because rail routes are physically limited by geographical features (e.g., mountains, water bodies) in ways that air routes are not. Indeed, the only way in which flight miles can be less than rail miles is a consequence of relative locations of airports and rail stations for a given city pair. Figure 4 shows rail miles plotted as a function of flight miles for the city pairs analyzed here. Based on the conclusions of previous studies noted above that found rail travel to have an advantage over air travel for distances less than 300–600 miles (500–1000 km), trips with rail distances of more than 500 miles are distinguished from those less than 500 miles in Figure 4.

The trend line, calculated from all city pair data, indicates that rail distance is approximately 30% longer than flight distance [similar to the 35% increase reported by Prussi and Lonza (2018)], with individual differences being considerably greater. For instance, the flight distance between Charlotte and Cincinnati is 335 miles, but the rail distance is 752 miles, about 2.25 times the flight distance. Beyond the issue of travel time, this has important implications for using rail to reduce travel-related CO_2_ emissions.

**Figure 5.**
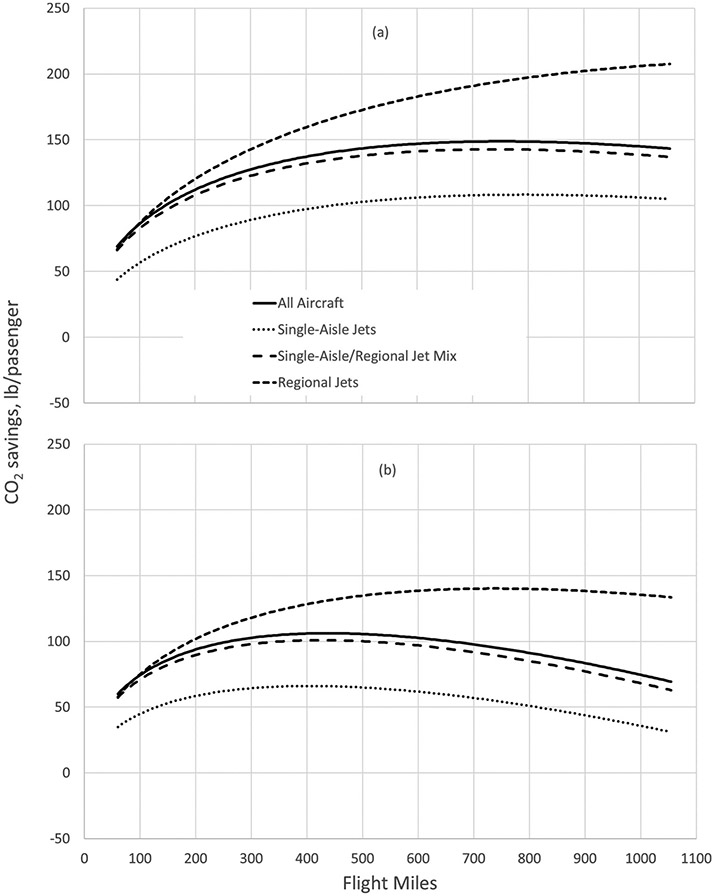
CO_2_ savings per passenger by flight miles and aircraft type. (a) Emissions savings assuming equivalent flight and rail miles. (b) emissions savings using adjusted rail miles.

These data show that, for the northeastern U.S. on average, longer rail distances reduce the CO_2_ advantage of rail by about 30% per mile of flight distance. The implications can be seen by comparing the plots in Figure 5. The top plot shows the average CO_2_ per passenger saved by rail travel compared to air travel in the northeastern U.S., assuming travel miles for air and rail are equivalent. The bottom plot accounts for the longer rail travel distance and indicates that the CO_2_ savings are greatest for flight distances of about 400 miles when comparing to all aircraft types. As flight distances increase and the corresponding rail distances increase even more, the CO_2_ savings of rail travel decrease, but remain positive for flight distances of more than 1100 miles, considerably beyond the range of the north-eastern U.S. being evaluated here.

### Comparison to different rail power

**Figure 6.**
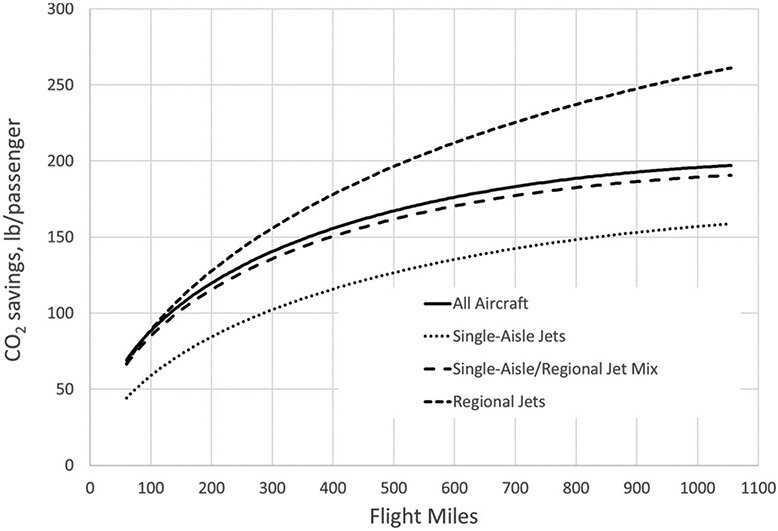
CO_2_ emissions savings by flight distance and aircraft type in lb/passenger for electric-powered rail, using adjusted rail miles.

As illustrated above in the development of rail emission factors for electric and diesel locomotives, the amount of CO_2_ emissions saved by rail travel compared to air travel in the northeastern U.S. is strongly dependent upon whether the rail line is electrified or not. The emissions savings presented in Figure 5 used a mileage-weighted average emission factor for travel on the Amtrak routes analyzed here. Looking only at the electrified portion of the Amtrak system, all of which are in the northeastern U.S., travel by rail yields CO_2_ savings over air travel regardless of aircraft type or length of trip, and accounting for higher rail miles compared to flight miles, as seen in Figure 6. Although Figure 6 presents emissions savings for route distances of up to 1000 flight miles, the longest all-electric trip that can now be made on Amtrak is 457 rail miles, from Boston to Washington (about 390 flight miles, depending upon which Washington-area airport is used).

**Figure 7.**
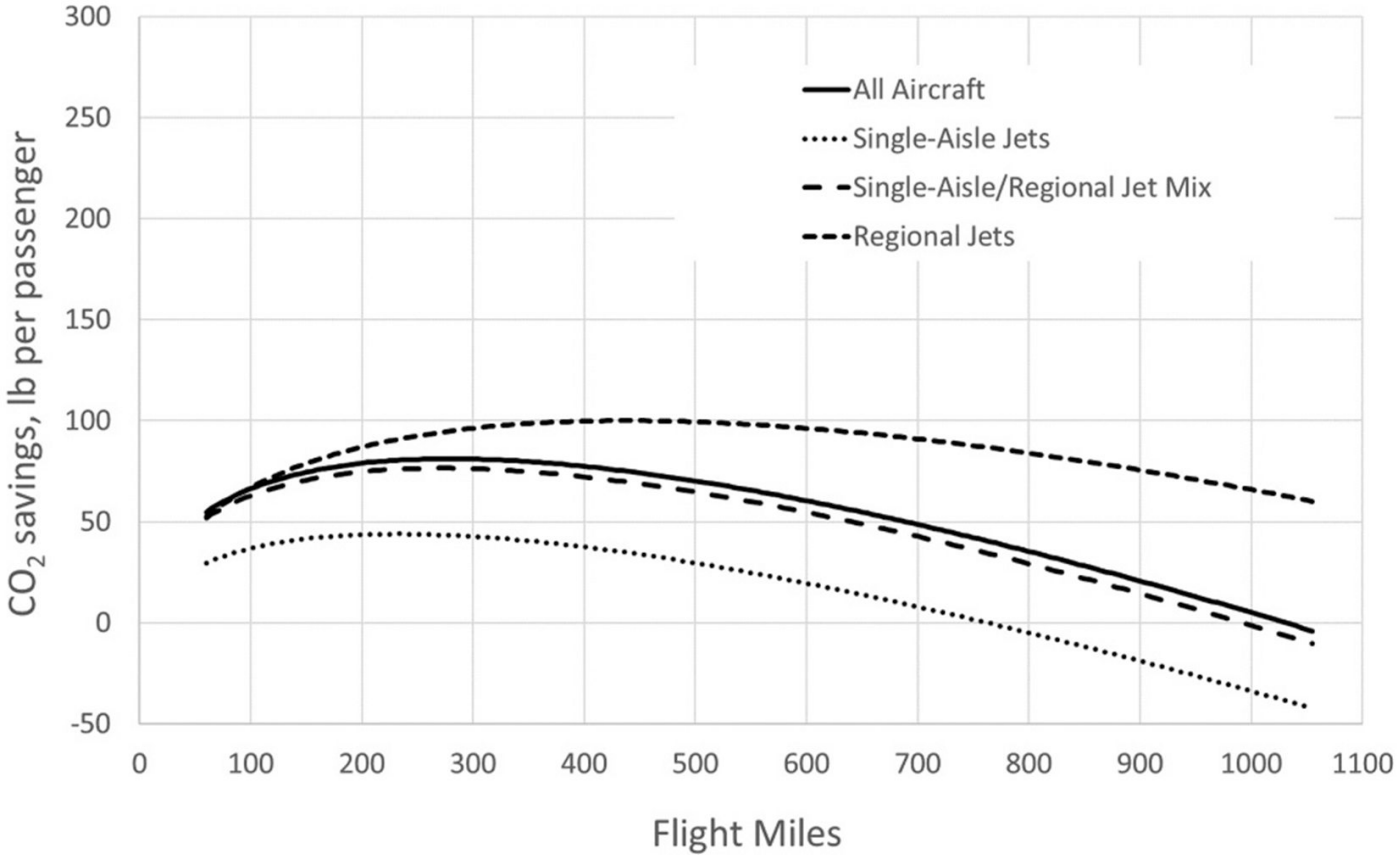
CO_2_ emissions savings by flight distance and aircraft type in lb/passenger for diesel-powered rail, using adjusted rail miles.

The situation for the non-electrified portions of Amtrak’s system is considerably different, as can be seen in Figure 7. Comparing air travel to rail travel over route segments that rely on diesel locomotives results in much reduced savings and a smaller range over which savings occur. For routes that operate with single-aisle jets, air travel generates less CO_2_ per passenger for trips longer than about 750 flight miles.

### Results summary

**Figure 8.**
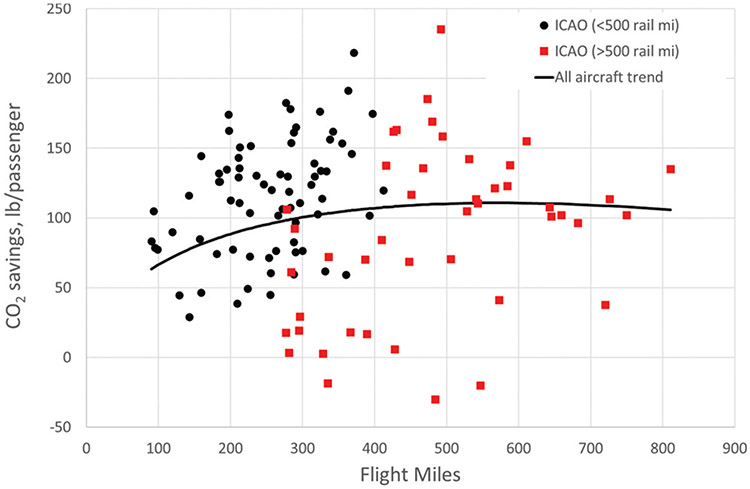
Comparison of CO_2_ emissions savings from ICAO emissions data to emissions savings estimates from this analysis for all city/airport pairs, using mile-weighted average rail emissions and all-aircraft flight emissions.

Saved CO_2_ estimates using the trend for all aircraft and all rail routes analyzed for the 120 ICAO airport pairs analyzed here, accounting for differences in travel distance, are compared in Figure 8 to the ICAO data. Results for rail distances greater than 500 miles are shown in the red squares. All city pairs for which CO_2_ emissions savings are negative (i.e., CO_2_ emissions are higher for rail travel than for air travel) occur for rail trips longer than 500 miles. Table 2 shows the numerical values for CO_2_ emissions savings in lb/passenger for the city pairs. City pairs for which the rail distance is greater than 500 miles are shown in shaded cells.

**Figure 9.**
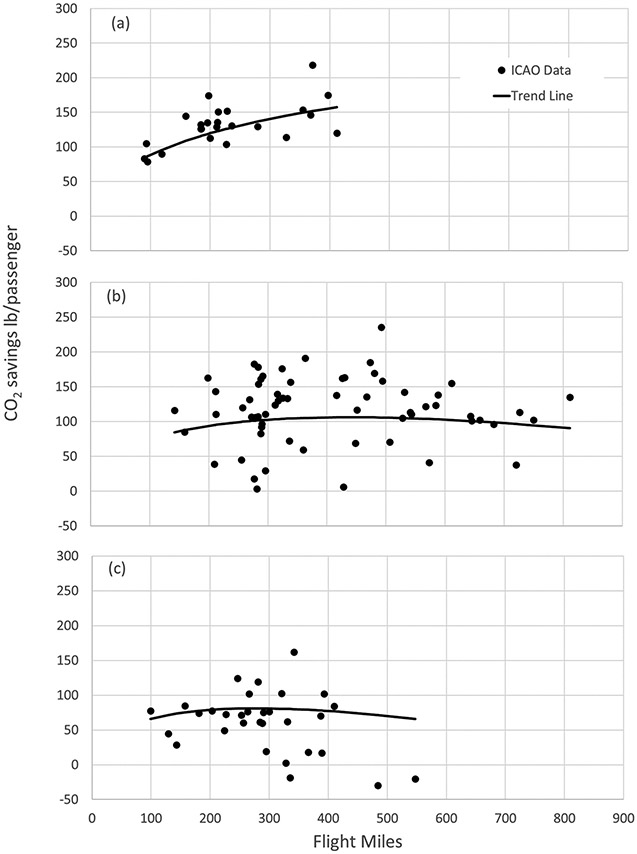
Comparison of CO_2_ emissions savings from ICAO emissions data to emissions savings estimated from this analysis for (a) city/airport pairs with electric rail; (b) city/airport pairs with mixed electric and diesel rail; and (c) city/airport pairs with diesel rail, using all-aircraft flight emissions.

The scatter in the data makes it difficult to describe with a single trend line. However, Figure 9 shows somewhat more consistent correlations between the trend lines and the ICAO data by comparing trends for electric-only, mixed electric/diesel, and diesel-only to their respective ICAO data points. Comparison to rail power type captures the general trends illustrated in Figures 6 and 7: CO_2_ savings increase with travel distance for electrified rail travel and are flat or beginning to decrease for travel on routes that rely on diesel power.

## Discussion

This relatively straightforward analysis illustrates a few key issues when comparing current per-passenger CO_2_ emissions savings on a single air vs. rail trip between cities in the northeastern U.S. First, it shows the significance of electrified rail as a means to reduce CO_2_ emissions due to intercity travel. As seen in Figures 5 and 6, electrified rail’s advantage in CO_2_ savings between electrified rail and diesel rail grows with increasing travel distance for distances within the range of this analysis. This advantage of electrified rail will only increase as the share of low-CO_2_ electricity generation continues to increase.

The second key issue is that the type of rail power is only part of the picture. Although flight emission factors show a fairly consistent trend as a function of flight distance for each aircraft category (as shown in Figure 3), the trends in CO_2_ savings by using rail as an alternative are much less clear, as seen in Figure 8. Different airport and flight segment characteristics, type of aircraft used, and differences between flight and rail distance all affect CO_2_ savings for a given city pair, to the extent that air travel can be the lowest-CO_2_ option in some cases, even for flight distances as short as 400 miles.

There are important limitations to this analysis, which also illustrate potential research needs and opportunities. First, it needs to be recognized that the underlying ICAO data for CO_2_ emissions between individual airport pairs are also estimates rather than measurements. The analysis here is derived from the ICAO emissions estimates and cannot be interpreted as an evaluation of those estimates, which provide averages and are not intended to convey day-to-day changes in passenger and freight loads, aircraft, weather, and other factors that affect fuel consumption and emissions for each flight. More generally, the need to use estimated emissions shows the difficulties in quantifying transportation CO_2_ emissions at resolutions finer than city-, system-, or nation-wide.

Second, the only available data for rail emissions are at a national average level, although the limited range of electrified intercity rail means that it is more immediately applicable to the current study scope. As rail travel grows in importance, higher resolution energy and emissions data will become increasingly helpful to understanding the relative advantages and disadvantages of intercity travel modes. For electric rail, allocating per-passenger emissions for a given train will require an approach that accounts for the different electricity generation mixes along a train’s route as well as changes in passenger load between stations and at different times.

Third, this analysis evaluates limited airport pairs and rail routes, largely located in the northeastern U.S. It is not appropriate to apply these results to the U.S. intercity transportation system more generally. Evaluation of intercity travel in other regions can add to these results; the region centered on Chicago and routes in California offer the most immediate opportunities for further study.

Fourth, other modes of intercity travel, such as buses or private passenger vehicles, are not considered. A full evaluation of CO_2_ emissions associated with intercity travel must include the full suite of options and their relative advantages and disadvantages.

Finally, this study does not consider costs, travel time, or transport between terminals and the ultimate travel end points. The analysis also does not consider the energy or emissions from airport ground operations, train switching, or other indirect operations, and does not account for upstream emissions associated with each energy source, or the life cycle emissions more generally. All of these are important factors in understanding CO_2_ emissions associated with intercity travel but are outside the scope of this work.

## Conclusion

Using readily accessible data, this analysis has evaluated the per-flight savings in CO_2_ emissions per passenger that are currently possible when traveling by rail rather than air in the northeastern U.S. The analysis showed considerable CO_2_ savings by using existing electrified rail as an alternative to air travel and that savings vary substantially with aircraft type and difference in rail and flight distances for a given city pair.

In addition to presenting this information for the existing air and rail travel infrastructure, the analysis has identified several data needs and research opportunities. There is a lack of route-specific data on emissions and energy use for passenger rail, which contrasts with the multiple, publicly oriented CO_2_ estimators for air travel. Understanding the broader policy implications of increasing rail travel as a means to reduce CO_2_ emissions will depend upon better and higher-resolution data. In addition, expanding the analysis to other regions of the Amtrak network can help identify differences and similarities that can be of value in developing national and regional policy options.

## Figures and Tables

**Table 1.** Cities, airports, and Amtrak stations included in the analysis.

City	Airport Code(s)	Amtrak Station Code	City	Airport Code(s)	Amtrak Station Code
Albany, NY	ALB	ALB	Philadelphia, PA	PHL	PHL
Baltimore, MD	BWI	BAL	Pittsburgh, PA	PIT	PGH
Boston, MA	BOS	BOS	Portland, ME	PWM	POR
Buffalo, NY	BUF	BUF	Providence, RI	PVD	PVD
Charlotte, NC	CLT	CLT	Raleigh, NC	RDU	RGH
Cincinnati, OH	CVG	CIN	Richmond, VA	RIC	RIC
New York, NY	EWR	NYP	Rochester, NY	ROC	ROC
	JFK				
	LGA		Springfield, MA	BDL	SPG
Norfolk, VA	ORF	NFK	Washington, DC	DCA	WAS
				IAD	

**Table 2.** Savings in CO_2_ per passenger traveling one way by rail instead of flight, lb CO_2_/passenger. NF indicates no direct flight between the city pair. Shaded cells indicate rail distances greater than 500 miles.

Rail		BOS	POR	PVD	SPG	NYP			PHL	BAL	WAS		RIC	NFK	RGH	CLT
	Airport	BOS	PWM	PVD	BDL	JFK	LGA	EWR	PHL	BWI	DCA	IAD	RIC	ORF	RDU	CLT
POR	PWM	NF	-													
PVD	PVD	NF	NF	-												
SPG	BDL	NF	NF	NF	-											
NYP	JFK	126	107	NF	NF	-										
	LGA	126	132	NF	NF	NF	-									
	EWR	113	178	144	NF	NF	NF	-								
PHL	PHL	130	191	131	135	105	79	NF	-							
BAL	BWI	146	117	114	107	132	NF	NF	83	-						
WAS	DCA	175	169	154	124	136	151	174	90	NF	-					
	IAD	120	235	219	134	104	152	129	NF	NF	NF	-				
RIC	RIC	185	NF	NF	NF	161	165	183	163	NF	NF	78	-			
NFK	ORF	136	NF	NF	NF	97	111	154	111	46	29	85	NF	-		
RGH	RDU	155	NF	NF	142	162	163	138	72	45	72	49	NF	NF	-	
CLT	CLT	114	135	96	108	114	111	105	69	59	62	103	60	60	45	-
CIN	CVG	102	NF	38	102	138	123	121	70	6	84	70	NF	−30	17	−18
PGH	PIT	159	NF	NF	NF	157	134	130	102	39	77	74	NF	NF	3	18
ALB	ALB	NF	NF	NF	NF	NF	NF	116	143	83	139	176	NF	NF	NF	101
ROC	ROC	162	NF	NF	NF	76	71	124	120	18	29	92	NF	NF	NF	41
BUF	BUF	102	NF	NF	NF	76	75	119	106	4	19	61	NF	NF	NF	−20
